# Non-dissemination in qualitative health research—A retrospective cohort study of conference abstracts

**DOI:** 10.1017/rsm.2026.10085

**Published:** 2026-04-14

**Authors:** Marwin Weber, Markus Toews, Waldemar Siemens, Andrew Booth, Simon Lewin, Heather Menzies Munthe-Kaas, Claire Glenton, Jane Noyes, Joerg J. Meerpohl, Ingrid Toews

**Affiliations:** 1 Institute for Evidence in Medicine, Medical Center – University of Freiburg / Medical Faculty – University of Freiburg, Freiburg, Germany; 2 Division of Population Health, School of Medicine and Population Health, Sheffield Centre for Health and Related Research, University of Sheffield, Sheffield, UK; 3 Centre for Epidemic Interventions Research, Norwegian Institute of Public Health, Oslo, Norway; 4 Department of Health Sciences, Norwegian University of Science and Technology, Ålesund, Norway; 5 Health Systems Research Unit, South African Medical Research Council, Cape Town, South Africa; 6 Department of Health and Functioning, Western Norway University of Applied Sciences, Bergen, Norway; 7 School of Health Sciences, Bangor University, Bangor, UK; 8 Cochrane Germany, Cochrane Germany Foundation, Freiburg, Germany

**Keywords:** cohort study, dissemination bias, GRADE-CERQual, publication bias, qualitative evidence synthesis, qualitative research

## Abstract

Dissemination bias can occur when qualitative research is published selectively, potentially reducing the confidence in qualitative evidence. This retrospective cohort study aims to quantify the extent of non-dissemination of qualitative health research by following 1,123 conference abstracts. The proportion of non-dissemination, the time to publication, as well as associations between author or study characteristics and full publication were examined. For 22.8% of these studies, no full publication could be identified within at least 6 and up to 8 years after their presentation. For those that were published, median time to publication was 11 months (95% CI 10 to 12). Studies from authors affiliated with institutions in Australia were more likely to be published than those from North America (OR 4.47; 95% CI 1.58 to 18.74). Oral presentations were more likely to be published than poster presentations (OR 3.40; 95% CI 1.57 to 8.20). Studies that used two qualitative data collection methods were more likely to be published than studies that used one qualitative method only (OR 1.53; 95% CI 1.01 to 2.38). Conference abstracts that reported no funding were less likely to be published than those which reported funding (OR 0.71; 95% CI 0.51 to 0.99). Publicly funded research was more likely to be published than privately funded research (OR 2.24; 95% CI 1.16 to 4.28). Given the considerable proportion of unpublished health-related qualitative studies, there is a reason to believe that dissemination bias may impact negatively on qualitative evidence synthesis. This can, in turn, impair decision-making that uses qualitative evidence.

## Highlights

### What is already known?

- Around 50% of all quantitative research studies remain unpublished. The proportion of non-publication in qualitative research was only investigated in small studies.
- Dissemination bias occurs when the non-publication of research is selective and based on the findings and main messages, potentially reducing the certainty in evidence syntheses. While this phenomenon is well-documented within quantitative research, its extent in qualitative health research remains unclear.

### What is new?

- In this retrospective cohort study, we followed 1,123 conference abstracts across various medical and health-related disciplines.
- Almost one quarter were still unpublished 6–8 years later. Half of the studies that were published after the conference were published after 11 months.

### Potential impact for RSM readers

- Many qualitative studies are never published. This represents research waste and missed opportunities to understand non-dissemination and dissemination bias in qualitative research and to develop methods to aid the assessment of the plausible impact of dissemination bias on qualitative evidence syntheses.

## 1 Background

Dissemination bias describes a systematic error occurring from the selective non-dissemination of studies and individual findings.^1^ In the context of qualitative research, dissemination bias has been defined as a systematic distortion of the phenomenon of interest due to selective dissemination of qualitative studies or the findings of qualitative studies.^2^ Also referred to as publication bias, dissemination bias involves more than the non-publication of studies. Dissemination bias considers when, where and in what format research or individual findings are published and covers underlying mechanisms or biases that impact on the accessibility of a study.^1^ Given its evidently harmful impact on quantitative research,^3^ ^,^ ^4^ it is pertinent to consider dissemination bias in qualitative research as well, where the extent of, reasons for and implications of this phenomenon are, as of yet, relatively uncharted.^2^

Until 2016, only one study had investigated non-dissemination in qualitative research.^5^ In 2016, a large international cross-sectional survey gathered data regarding non-dissemination and dissemination bias in qualitative research from the scientific community.^6^ Notably, almost 70% of the participating researchers reported that at least one of their qualitative studies had not been published in a peer-reviewed journal. Respondents perceived that non-dissemination of qualitative research has negative impacts on health policy. In a further study, a cohort of qualitative studies was followed.^7^ The authors found that one-third of qualitative studies presented at nursing research conferences did not result in full publication (including full texts in scientific journals as well as in grey literature) 5 years after presentation. Previous literature had supported the notion that dissemination bias in qualitative research is likely to lead to poor decision-making, potential research waste, and missed opportunities to support healthcare decisions, planning, and implementation.^8^ Dissemination bias has therefore been suggested as a candidate domain of the GRADE-CERQual approach for assessing confidence in findings from qualitative evidence syntheses.^2^ Still, there is insufficient evidence in this field to draw clear conclusions on its impact and consequences. The present study is linked to the development of the domain “Dissemination bias” in the GRADE-CERQual approach. It is intended to serve as a foundation for further research and conceptualization of the “Dissemination bias” domain within the overall approach. This study aims to extend the results of previous research by mapping and quantifying the extent of non-dissemination in qualitative health research, the time to publication and the factors associated with non-dissemination in a representative sample of qualitative studies in health and healthcare.

## 2 Methods

### 2.1 Study setting and data sources

Using a retrospective cohort study design, we followed a cohort of health-related qualitative studies initially presented as conference abstracts to investigate their full publication status later on. We used purposive, non-probabilistic sampling to build our study sample. We included studies that were presented at conferences between 2016 and 2018. We selected this time period to allow sufficient time for full publication. The follow-up period from conference presentation to the search for full publication was 6–8 years. Study methods build on methods of a previous study^7^ and refined these based on peer-review feedback. The study is reported according to the STROBE statement^9^ and registered in OSF (https://osf.io/u7gtw).^10^

Abstracts were retrieved via searches in Web of Science Conference Proceedings Citation Index – Science (CPCI-S) and Conference Proceedings Citation Index – Social Science & Humanities (CPCI-SSH) via the Web of Science Platform (Clarivate) on September 21, 2022 (see Supplement 1 of the Supplementary Material for search strategy). We selected this database due to its large coverage of conference abstracts in health and healthcare research. The search strategy was based on validated search strategies for related databases and adapted for this study.^11^ ^,^ ^12^

To be included, abstracts needed to fulfill one of the following criteria: (i) focused on describing an original qualitative study related to health and healthcare, (ii) described a mixed-methods study or a program description with at least one qualitative element, or (iii) described an evidence synthesis of qualitative research findings. We excluded abstracts reporting exclusively quantitative studies, broad methodological or theoretical discussions. Also, studies lacking a clear focus on health and healthcare research were excluded. A lack of reporting detail was not considered as a reason for exclusion. See Supplement 2 of the Supplementary Material for further information on inclusion and exclusion criteria of studies.

To identify eligible studies, each abstract was screened by at least two independent reviewers. In total, eight reviewers (MT, AB, SL, HMK, CG, JN, IT, and JN) participated in the screening process in Covidence.^13^ We extracted author information and study characteristics (see Table 1) from the database record of all included studies by using a semi-automated approach with repeated, regular consistency checks in R-Studio.^14^ Data that could not be extracted by the semi-automated approach were extracted by two researchers (MW and CS) between February and August 2023. We focused only on selected data, that is, on the first author, to ensure better manageability of the data and analyses. This information was used to match full publications and, subsequently, to explore potential factors associated with dissemination. Multiple publications of one study were consolidated within a single unit of analysis and a full account of the reported information was considered in the data extraction and analyses.

**Table 1:** Sample characteristics Table 1 Long description.

Sample characteristics	*n* (%)
Included studies	1,123 (100)
Presentation format	
Presentation	70 (6.23)
Poster	233 (20.75)
Unclear	820 (73.02)
Conference location	
North America	530 (47.2)
Europe	452 (40.25)
Asia	109 (9.71)
Australia	27 (2.4)
South America	4 (0.36)
Africa	1 (0.09)
Author characteristics	
Gender of 1st author	
Female	794 (70.7)
Male	316 (28.14)
Unclear	13 (1.16)
Affiliation location	
North America	460 (40.96)
Europe	445 (39.63)
Asia	137 (12.2)
Australia	41 (3.65)
Africa	15 (1.34)
South America	15 (1.34)
Not reported	10 (0.98)
Affiliation category	
Public	1,070 (95.28)
Private	23 (2.05)
Not reported	30 (2.85)
Study characteristics	
Number of population groups included	
1	864 (76.94)
2	180 (16.03)
3	55 (4.9)
4	8 (0.71)
5	2 (0.18)
Not reported	14 (1.25)
Number of qualitative data collection methods used	
1	882 (78.54)
2	169 (15.05)
3	34 (3.03)
4	6 (0.53)
Not reported	32 (2.85)
Level of healthcare addressed	
Tertiary care	298 (26.54)
Community care	204 (18.17)
Secondary care	162 (14.43)
Primary care	106 (9.44)
Mixed	42 (3.74)
Unclear	311 (27.69)
Funding reported	
No	798 (71.06)
Yes	325 (28.94)
Funding type	
Public / non-profit	242 (21.55)
Private / for-profit	78 (6.95)
No funding	5 (0.45)
Not reported	798 (71.06)
Study aim reported	
Yes	1,039 (92.52)
No	84 (7.48)
Study conclusions reported	
Yes	1,038 (92.43)
No	85 (7.57)
Study type	
Cross sectional	1,006 (89.58)
Longitudinal	98 (8.73)
Not reported	19 (1.69)

### 2.2 Searching for full publications and matching

We used two approaches, a survey and systematic literature searches, to identify full publications of the studies initially presented as conference abstracts. For the survey, contact information extracted from the database record was used to contact the study authors. The survey was created and distributed via personalized e-mail using the SoSci Survey^15^ platform to ascertain a full publication of the respective studies. Detailed results of the survey are published elsewhere.^16^

For the literature searches, Google Scholar; MEDLINE via OVID; ProQuest & Google were searched in a stepwise approach for full publications/texts of the included abstracts. We conducted the searches between August 2023 and August 2024. We used individual search strategies for each study using a combination of author names, text words describing the phenomenon of interest, and the study methods as reported in the conference abstract. We combined search concepts using Boolean operators (AND and OR) and the applicable database settings, respectively, to find candidate publications. In case of either zero or too many (>20) search hits, we broadened or narrowed the search strategy by searching exact phrases and/or adapting the Boolean operators. Potentially matched full publications were screened for eligibility against author- and study information. We considered a full publication a match if the phenomena of interest and the method(s) of inquiry were the same in both records, and at least one of the conference abstract authors were authors in the full publication.

We considered a full publication to be any form of full text adding substantial detail to the original conference abstract information and reporting methods, final findings and to the interpretation or discussion of the findings. Substantive full publications included journal articles (both peer-reviewed and not peer-reviewed and extended versions published as conference proceedings, brief reports, and research letters), books, and book chapters (including collections of conference proceedings), grey literature (theses or dissertations published by organizations outside traditional commercial or academic publishing) and any other form of extended versions (e.g., conference proceedings published via standalone electronic libraries, conference organizers or universities, letters). For each matched pair of outputs, we extracted the month and year of publication and type of publication for the full publication.

### 2.3 Statistical analysis

All study data were documented in MS Excel 2016 and analyzed with R.^14^ First, data were summarized with univariate descriptive statistics. The proportions of studies with and without full publication were analyzed. Fisher’s exact test was calculated to examine associations between dichotomous study- and author characteristics and the proportion of abstracts with a matching full publication. Logistic regression analyses were used to analyze associations between differently scaled (categorical and ordinal) study- and author characteristics (see Supplement 3 of the Supplementary Material for further details) and full publication. For ordinal scaled categories, we selected the factor with the most cases as reference post hoc in the logistic regression. When testing for associations, we excluded unspecific categories (e.g., unclear; not reported) in order to be able to assign associations more clearly. Results are reported as odds ratios (ORs) together with their 95% confidence intervals (CIs).

Median time to publication was calculated with a time-to-event analysis and illustrated using a cumulative incidence plot. For time-to-event analyses, when the exact conference month was not reported but the year was known, we substituted December for the missing value as the most conservative estimate. Additionally, we evaluated associations of study and author characteristics with duration to publication using Cox regression analyses (R packages survival^17^ and survminer^18^). All analyses were bivariate, focusing on exploring simple associations between a single independent variable and the dependent variable. Dichotomous data are presented as frequencies and proportions, while continuous data are summarized as means and standard deviations (SDs) or as medians, depending on the distribution of the data.

## 3 Results

**Figure 1:**
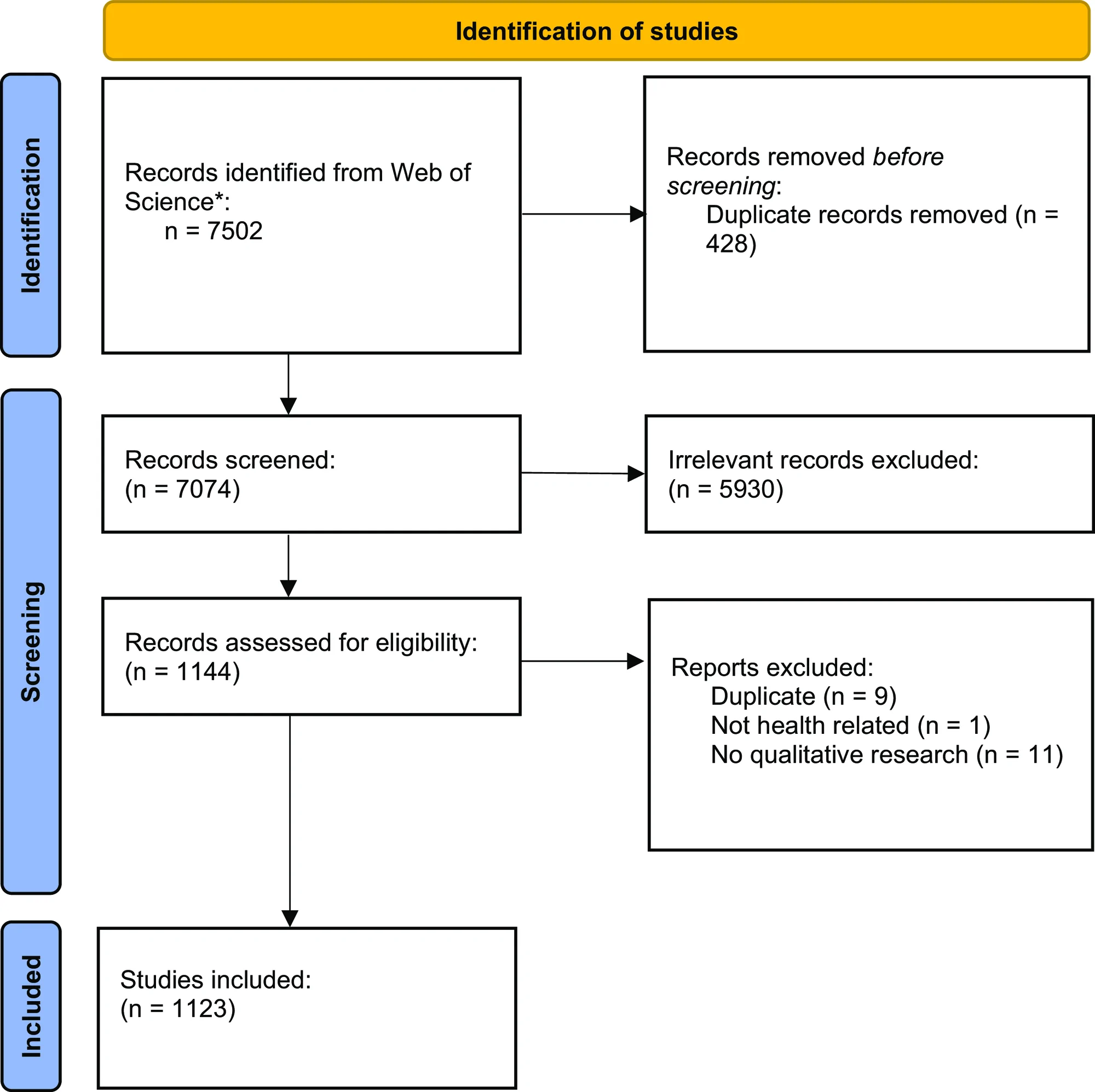
Flow chart of conference abstracts and follow-up. Figure 1 Long description.

We found 7,502 records in our search. A total of 428 records were automatically excluded as duplicates. After screening 7,074 records, we included 1,123 conference abstracts for follow-up. A total of 5,951 records were excluded because they did not fulfill our eligibility criteria (see Figure 1). The majority of the included studies were presented at conferences in North America or Europe. The majority of the abstracts’ first authors were female. Most authors were affiliated with a North American or European institution. In most included studies, data were retrieved from a single population group and most studies employed one method of data collection. About one-third of studies reported that they had received funding, of which approximately three-quarters were publicly funded. Almost all of the abstracts reported the study aim and conclusions. We defined the majority of the studies as cross-sectional rather than longitudinal. See Table 1 for more detailed information.

### 3.1 Proportions of non-dissemination and time-to-publication

**Figure 2:**
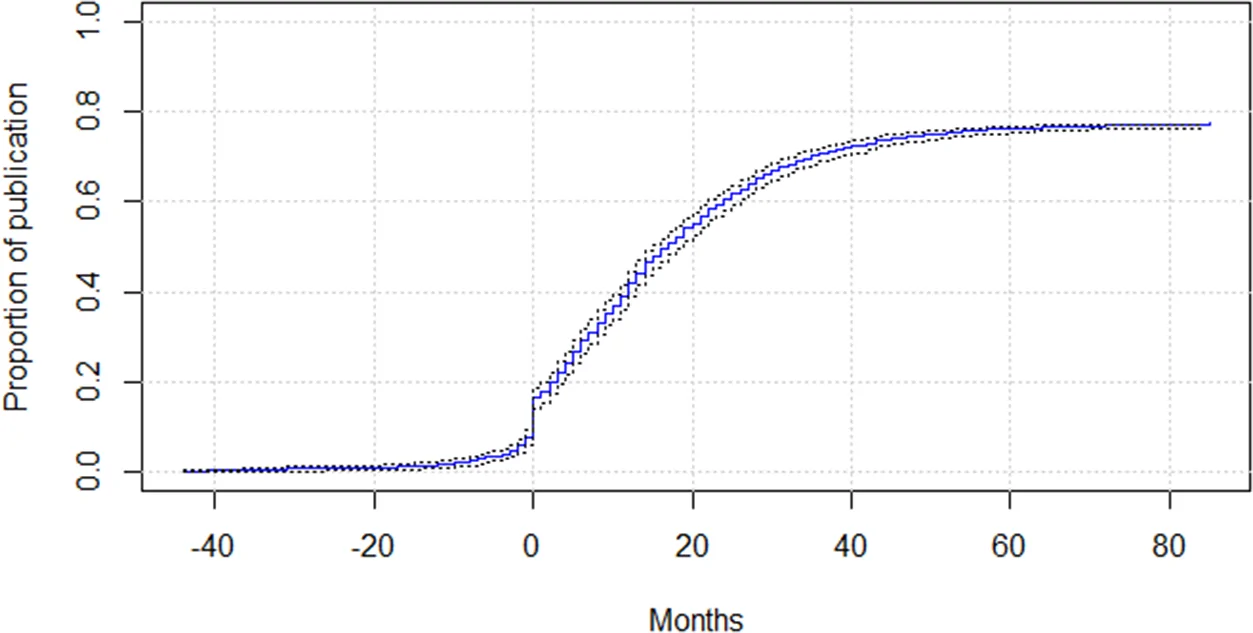
Cumulative incidence plot with 95% CIs illustrating the time to full publication of qualitative studies presented at conferences. *Note*: Due to the full publication rate of 0.77, the median is visualized at 0.385 on the scale for proportion of publication in this cumulative incidence plot. “0” on the scale for months represents the conference date. Figure 2 Long description.

For 256 of the 1,123 (22.80%) included conference abstracts, no full publication could be retrieved within at least 6 (for studies presented in 2018) and up to 8 years (for studies presented in 2016) after presentation. Conversely, for 867 of 1,123 (77.20%), we retrieved at least one matching full publication. We retrieved 113 of the 867 matching full publications (13.03%) via the survey. Of all full publications, 642 (74.05%) were published in a scientific journal, 35 (4.04%) in a book or book chapter, 27 (3.11%) in grey literature, and 163 (18.8%) as other publication format. The median time to any full publication was 11 months (95% CI 10 to 12 months) (see Figure 2).

### 3.2 Characteristics associated with dissemination

Table 2 contains results for associations of the conference abstract characteristics with full publication. We found that studies from authors affiliated with institutions in Australia were more than four times more likely to result in a full publication than those from North America (OR 4.47; 95% CI 1.58 to 18.74). Other author characteristics showed no statistically significant associations.

**Table 2:** Conference abstract characteristics and their association with full publication Table 2 Long description.

Factor	OR (95% CI)
Author characteristics	
Gender of 1st author (male vs. female)^a^	1.2 (0.86–1.69)
Affiliation location^b^	
North America (reference category)	Not applicable
Europe	1.34 (0.98–1.82)
Africa	2.29 (0.62–14.81)
South America	1.41 (0.44–6.27)
Asia	1.31 (0.84–2.11)
Australia	4.47 (1.58–18.74)*
Affiliation category (public vs. private)^a^	1.24 (0.40–3.34)
Study characteristics	
Presentation format (presentation vs. poster) ^a^	3.40 (1.57–8.2)*
Number of population groups included ^b^	
1 (reference category)	Not applicable
2	0.78 (0.54–1.13)
>2	1.03 (0.58–1.98)
Number of qualitative data collection methods used^b^	
1 (reference category)	Not applicable
2	1.53 (1.01–2.38)*
>2	1.27 (0.6–2.99)
Level of healthcare addressed^b^	
Tertiary care (reference category)	Not applicable
Secondary care	1.00 (0.65–1.58)
Primary care	1.09 (0.65–1.87)
Community care	1.39 (0.90–2.18)
Mixed	0.80 (0.40–1.69)
Funding reported (yes vs. no) ^a^	0.71 (0.51–0.99)*
Funding type (public / non profit vs. private / for profit) ^a^	2.24 (1.16–4.28)*
Study aim reported (yes vs. no) ^a^	1.14 (0.65–1.94)
Study conclusions reported (yes vs. no) ^a^	1.15 (0.65–1.94)
Study type (cross-sectional vs. longitudinal) ^a^	1.47 (0.89–2.36)

*Note*: Missing data and unclear information were excluded for the analysis of associations.

Abbreviations: OR, odds ratio; CI, confidence interval.

a Calculated with Fisher’s exact test.

b Calculated with logistic regression.

* *p* < 0.05.

The following study characteristics showed statistically significant associations with a full publication: Oral presentations were over three times more likely to result in a full publication than poster presentations (OR 3.40; 95% CI 1.57 to 8.20). Studies that used two qualitative data collection methods were one and a half times more likely to result in a full publication than studies that used one qualitative method only (OR 1.53; 95 CI 1.01 to 2.38). Conference abstracts that reported no funding were less likely to result in a full publication as those which reported funding (OR 0.71; 95% CI 0.51 to 0.99). Publicly funded research was over twice as likely to result in a full publication when compared to privately funded research (OR 2.24; 95% CI 1.16 to 4.28).

Other study characteristics showed no statistically significant associations.

### 3.3 Characteristics associated with time-to-publication

We found that studies from authors affiliated with institutions in Australia and Asia were associated with shorter time to publication. Furthermore, oral presentation as presentation format, community care as level of care, reporting of the funding as well as not reporting of study aim, and study conclusion showed associations with shorter time-to-publication. Other study or author characteristics showed no statistically significant associations with time-to-publication. Full results and plots of the Cox Regression are presented in the Supplement 4 of the Supplementary Material.

## 4 Discussion

We conducted a retrospective cohort study to investigate the subsequent full publication of qualitative health- and healthcare-related studies initially presented as conference abstracts. We found that for almost one-quarter (22.8%) of conference abstracts no full publication could be retrieved after up to 8 years. Half of the included studies resulted in a full publication within around 11 months after the conference. The presentation format, geographic location of the first authors’ affiliation, number of methods used for data retrieval, reporting of the funding as well as funding type seemed to be associated with a full publication. Moreover, Australian and Asian affiliated authorship, presentation as presentation format, community care as level of care, reporting of the funding as well as not reporting of study aim, and study conclusion seemed to be associated with a shorter time-to-publication.

This study showed lower proportions of non-dissemination than previous studies with a similar research question. A study^7^ with a similar design of qualitative research initially presented at a nursing conference showed a slightly higher proportion of no subsequent full publications (33.7%). In another study of qualitative research, Petticrew et al.^5^ uncovered a non-publication proportion of 55.8% of abstracts. Both studies had slightly shorter follow-up timeframes (5–7 years) than our present study. To contextualize our findings, in quantitative health-related research, the proportion of published studies ranged between 45.8% and 53.8% with a follow-up time between at least 2 and up to 10 years.^19^ ^,^ ^20^

These differences might be explained by more exhaustive search methods and structural improvements in research publishing. We conducted extensive follow-up searches in multiple databases and grey literature and sought direct author contact to obtain full publications. We extended the duration of follow-up in comparison to other studies to up to 8 years. Furthermore, we drew our study sample from an electronic database with a broad thematic scope. Therefore, we included abstracts from different conferences with different abstract submission and presentation criteria. In our sample, a substantial part of full publications was published simultaneously with the conference in form of proceeding papers (see cumulative incidence plot in Figure 2), which we considered a full publication according to the previously defined inclusion criteria as they clearly reported results and exceeded the conference abstracts. We searched for publications in scientific and grey literature to capture different forms of publications of completed research, not just journal publications. All these factors might have increased the yield of our search for (subsequent) publications. Besides methodological factors that might have promoted a higher detection of subsequent publications, it can be hypothesized that the proportions of subsequent publications of conference abstracts have, in fact, increased. This will have to be investigated in future research.

Structurally, the research publication system has evolved in recent years increasingly encouraging registration of different types of research, using research protocols, thereby increasing scientific rigor.^21^ ^,^ ^22^ This might be reflected in a higher conversion rate of studies presented as conference abstracts to full publications.

The time to publication (median 11 months) was similar to the previous study of Toews et al.^7^ For comparison, a corresponding study of clinical trials^23^ showed that median time to publication of clinical trials was 4.8 years from enrolment of the first participant and 2.1 years from trial completion. The phenomenon of qualitative studies being published before the conference date was also observed in Toews et al.^7^ as well. Similar observations have been reported in specific clinical fields, for example, in pediatric radiology^24^ and pediatric surgery,^25^ and forensic imaging,^26^ although this aspect has typically not been analyzed systematically across research areas. Consequently, direct comparisons between disciplines remain limited.

In other (health-related) areas, such as medical and health informatics, assistive technologies, or bioinformatics, we found that the full publication of results as conference paper is more common or even required by the conference submission guidelines. Regarding the associations, oral presentations compared to poster presentations were also found to be associated with higher chances of full publication as well as time-to-publication in a study^7^ with a similar methodological approach. Other significant associations did not correspond with the previous study.^7^ Due to the exploratory approach, we are not able to draw final conclusions, and advise against doing so.

## 5 Limitations

We drew a large, representative, and systematic sample from a search in Web of Science—a database that contains the highest number of conference abstracts in health and healthcare research. In comparison to other studies on this topic that drew samples from conference books and proceedings, sampling from a database is a novel and efficient approach that allows for more representative findings. Furthermore, we explicitly searched for full publications in the grey literature. We initially planned to search the EThOS database but it was not accessible during the search period. Therefore, we relied on Google searches for grey literature and did not search institutional repositories as this was too time consuming.

We followed a comprehensive process to match full publications with the relevant conference abstract. Nonetheless, matching through the phenomenon of interest, author, and qualitative method of inquiry was sometimes challenging, due to the brevity of reporting in conference abstracts.

Due to the large dataset and therefore feasibility, data extraction and searching for full publications and matching was not conducted in duplicate. A second study author (IT) checked all extracted study data for accuracy. The search strategies for full publications were randomly checked for accuracy and tested, likewise the matching full publications were checked. The latter two pertained to about 30% of the dataset.

We encountered issues of missing or unclear information (e.g., non-standardized reporting about the level of care or the methods used) that made it difficult to code study and author information clearly. This may have affected the validity of the respective associations between author and study characteristics and full publication. Missing information during the extraction and unclear cases during the matching were double-checked and discussed continuously throughout the process.

Moreover, we used study and author characteristics to investigate which factors might be associated with subsequent non-dissemination. The underlying bivariate analysis was inherently exploratory. As a result, confounder control or complex model selection procedures were not necessary. This approach may have potentially led to an overestimation of associations. Although we carefully selected characteristics, our analysis has multiple comparisons possibly resulting in false positive findings.

Overall, there is a chance that the proportions of non-dissemination of qualitative studies is substantially different from our findings. We were unable to account for those qualitative studies that were conducted but never presented at a conference. The impact of missing studies is likely to remain greater than studies omitted through sampling. In general, it is challenging and time consuming to establish the extent of non-dissemination from conference presentations without record linkage and transparent reporting. Mandating study identifiers for qualitative research, and requiring study protocols and/or a study registry for qualitative studies might enhance transparency and simplify matching to full publications.^21^ ^,^ ^27^ It is important to emphasize that this would not aim to solve non-dissemination or dissemination bias, but might help provide a clearer and more precise picture of non-dissemination and sensitize interest holders for the potential impact of dissemination bias.

We recommend that future studies carefully balance sample size with feasibility of a study, recognizing that extending the search scope or restricting abstract inclusion may still lead to underrepresentation. Studies may extend the coverage to more than one database while, at the same time, narrowing the inclusion criteria for the abstracts. A large sample of studies with standardized specifications may improve the structure and clarity of data extraction potentially allowing for a closer focus on the nature and content of the study findings which are of key interest for investigating dissemination bias. Nevertheless such methods remain exploratory in nature. Further insights of reasons for non-dissemination beyond those that are applicable to research in general,^6^ and that are related to the message and findings, remain of persistent interest if we are to understand more about actual dissemination bias in qualitative research.

## 6 Conclusions

Our study extends previous research, building a knowledge base for understanding the proportion of non-dissemination in qualitative research. We found that the proportion of non-dissemination in qualitative research is still considerable, too. Ascertaining the true proportion of non-dissemination is challenging as some studies may never be presented at conferences. We therefore assume that dissemination bias might potentially impact on qualitative evidence synthesis and thereby on decision-making that uses qualitative evidence.

Conceptually, using unpublished trials and systematic reviews of intervention effects as a reference, the impact of dissemination bias in qualitative research may be similarly grave on the confidence we may have in findings of evidence synthesis. Definitive data on dissemination bias remain elusive given the difficulties in identifying examples which can be tracked and the challenge of finding evidence for its existence, especially in qualitative research. In order to build a framework for understanding dissemination in qualitative research, more research is needed on how dissemination bias influences the qualitative evidence base and how we may begin to identify these distortions.

## Supporting information

10.1017/rsm.2026.10085.sm001 Weber et al. supplementary material Weber et al. supplementary material

## Data Availability

Data and analysis code are available in the Open Science Framework (OSF) at https://osf.io/36ypd. This study is registered in OSF at https://osf.io/u7gtw.

## Supplementary material

To view supplementary material for this article, please visit http://doi.org/10.1017/rsm.2026.10085.

## Long descriptions

### Table 1 Long description

The table is organized into two columns: Sample characteristics and n (%).

- Included studies: Total 1,123 (100%).

- Presentation format: Presentation 70 (6.23%), Poster 233 (20.75%), Unclear 820 (73.02%).

- Conference location: North America 530 (47.2%), Europe 452 (40.25%), Asia 109 (9.71%), Australia 27 (2.4%), South America 4 (0.36%), Africa 1 (0.09%).

- Author characteristics:

- Gender of 1st author: Female 794 (70.7%), Male 316 (28.14%), Unclear 13 (1.16%).

- Affiliation location: North America 460 (40.96%), Europe 445 (39.63%), Asia 137 (12.2%), Australia 41 (3.65%), Africa 15 (1.34%), South America 15 (1.34%), Not reported 10 (0.98%).

- Affiliation category: Public 1,070 (95.28%), Private 23 (2.05%), Not reported 30 (2.85%).

- Study characteristics:

- Number of population groups: 1 group 864 (76.94%), 2 groups 180 (16.03%), 3 groups 55 (4.9%), 4 groups 8 (0.71%), 5 groups 2 (0.18%), Not reported 14 (1.25%).

- Qualitative data collection methods: 1 method 882 (78.54%), 2 methods 169 (15.05%), 3 methods 34 (3.03%), 4 methods 6 (0.53%), Not reported 32 (2.85%).

- Level of healthcare: Tertiary care 298 (26.54%), Community care 204 (18.17%), Secondary care 162 (14.43%), Primary care 106 (9.44%), Mixed 42 (3.74%), Unclear 311 (27.69%).

- Funding: No 798 (71.06%), Yes 325 (28.94%).

- Funding type: Public / non-profit 242 (21.55%), Private / for-profit 78 (6.95%), No funding 5 (0.45%), Not reported 798 (71.06%).

- Study aim reported: Yes 1,039 (92.52%), No 84 (7.48%).

- Study conclusions reported: Yes 1,038 (92.43%), No 85 (7.57%).

- Study type: Cross sectional 1,006 (89.58%), Longitudinal 98 (8.73%), Not reported 19 (1.69%).

Navigate back to Table 1.

### Figure 1 Long description

The flowchart is organized into three vertical phases labeled on the left: Identification, Screening, and Included.

1. Identification Phase:

- Top-left box: Records identified from Web of Science* (n = 7502). An arrow points right to an exclusion box.

- Top-right box: Records removed before screening: Duplicate records removed (n = 428).

- A vertical arrow leads from the first box down to the Screening phase.

2. Screening Phase:

- Middle-left box: Records screened: (n = 7074). An arrow points right to an exclusion box.

- Middle-right box: Irrelevant records excluded: (n = 5930).

- A vertical arrow leads down to the next step.

- Lower-middle-left box: Records assessed for eligibility: (n = 1144). An arrow points right to an exclusion box.

- Lower-middle-right box: Reports excluded: Duplicate (n = 9), Not health related (n = 1), No qualitative research (n = 11).

- A vertical arrow leads down to the final phase.

3. Included Phase:

- Bottom-left box: Studies included: (n = 1123).

Navigate back to Figure 1.

### Figure 2 Long description

The horizontal X axis is labeled Months and ranges from minus 40 to 80 in increments of 20. The vertical Y axis is labeled Proportion of publication and ranges from 0.0 to 1.0 in increments of 0.2. A solid blue line represents the cumulative incidence, flanked by two black dotted lines representing the 95 percent C I s.

From minus 40 to 0 months, the proportion remains near 0.0 with a very slight upward curve starting around minus 10. At month 0, which represents the conference date, there is a sharp vertical step up to approximately 0.18. Following month 0, the curve shows a steep sigmoidal increase, passing the 0.4 proportion mark around month 15 and the 0.6 mark around month 25. The curve begins to plateau after month 40, reaching an asymptotic level of approximately 0.77 by month 60 and remaining stable through month 80. The confidence interval bands are narrowest at the start and end of the timeline and slightly wider during the period of rapid increase between months 0 and 40.

Navigate back to Figure 2.

### Table 2 Long description

The table consists of two columns: Factor and O R (95% C I).

Author characteristics:

* Gender of 1st author (male vs. female): 1.2 (0.86–1.69).

* Affiliation location: North America is the reference category. Europe: 1.34 (0.98–1.82). Africa: 2.29 (0.62–14.81). South America: 1.41 (0.44–6.27). Asia: 1.31 (0.84–2.11). Australia: 4.47 (1.58–18.74) with a p-value less than 0.05.

* Affiliation category (public vs. private): 1.24 (0.40–3.34).

Study characteristics:

* Presentation format (presentation vs. poster): 3.40 (1.57–8.2) with a p-value less than 0.05.

* Number of population groups included: 1 is the reference category. 2: 0.78 (0.54–1.13). Greater than 2: 1.03 (0.58–1.98).

* Number of qualitative data collection methods used: 1 is the reference category. 2: 1.53 (1.01–2.38) with a p-value less than 0.05. Greater than 2: 1.27 (0.6–2.99).

* Level of healthcare addressed: Tertiary care is the reference category. Secondary care: 1.00 (0.65–1.58). Primary care: 1.09 (0.65–1.87). Community care: 1.39 (0.90–2.18). Mixed: 0.80 (0.40–1.69).

* Funding reported (yes vs. no): 0.71 (0.51–0.99) with a p-value less than 0.05.

* Funding type (public / non profit vs. private / for profit): 2.24 (1.16–4.28) with a p-value less than 0.05.

* Study aim reported (yes vs. no): 1.14 (0.65–1.94).

* Study conclusions reported (yes vs. no): 1.15 (0.65–1.94).

* Study type (cross-sectional vs. longitudinal): 1.47 (0.89–2.36).

Navigate back to Table 2.
