# Supplementary material for: Non-dissemination in qualitative health research—A retrospective cohort study of conference abstracts
Source: Res Synth Methods. 2026 Apr 14;17(5):927–38. doi: 10.1017/rsm.2026.10085 (PMC13732349; doi:10.1017/rsm.2026.10085)
Supplement: Supplementary file 1 [file S1759287926100854sup001.docx]

Supplement 1 - Search strategy for Web of Science

(((AB=(((("semi-structured" or semistructured or unstructured or informal or "in-depth" or indepth or "face-to-face" or structured or guide) NEAR/2 (interview* or discussion* or questionnaire*))))) OR AB=((“focus group*” or qualitative or ethnograph* or fieldwork or "field work" or "key informant*"))

OR (TI=(interview* or “focus group*” or qualitative* or “grounded theory” or phenomenological or hermeneutic* or ethnogra* or “thematic analys*” or finding*))))

AND ((SJ==("AGRICULTURE" OR "ALLERGY" OR "ANATOMY MORPHOLOGY" OR "ANESTHESIOLOGY" OR "ANTHROPOLOGY" OR "AUDIOLOGY SPEECH LANGUAGE PATHOLOGY" OR "BEHAVIORAL SCIENCES" OR "BIOCHEMISTRY MOLECULAR BIOLOGY" OR "BIODIVERSITY CONSERVATION" OR "CARDIOVASCULAR SYSTEM CARDIOLOGY" OR "BIOTECHNOLOGY APPLIED MICROBIOLOGY" OR "BIOPHYSICS" OR "CELL BIOLOGY" OR "DENTISTRY ORAL SURGERY MEDICINE" OR "DERMATOLOGY" OR "DEVELOPMENTAL BIOLOGY" OR "EMERGENCY MEDICINE" OR "ENDOCRINOLOGY METABOLISM" OR "ENTOMOLOGY" OR "ENVIRONMENTAL SCIENCES ECOLOGY" OR "EVOLUTIONARY BIOLOGY" OR "FISHERIES" OR "FOOD SCIENCE TECHNOLOGY" OR "FORESTRY" OR "GASTROENTEROLOGY HEPATOLOGY" OR "GENERAL INTERNAL MEDICINE" OR "GENETICS HEREDITY" OR "GERIATRICS GERONTOLOGY" OR "HEALTH CARE SCIENCES SERVICES" OR "HEMATOLOGY" OR "IMMUNOLOGY" OR "INFECTIOUS DISEASES" OR "INTEGRATIVE COMPLEMENTARY MEDICINE" OR "LEGAL MEDICINE" OR "LIFE SCIENCES BIOMEDICINE OTHER TOPICS" OR "MARINE FRESHWATER BIOLOGY" OR "MATHEMATICAL COMPUTATIONAL BIOLOGY" OR "MEDICAL ETHICS" OR "MEDICAL INFORMATICS" OR "MICROBIOLOGY" OR "NEUROSCIENCES NEUROLOGY" OR "NURSING" OR "NUTRITION DIETETICS" OR "OBSTETRICS GYNECOLOGY" OR "ONCOLOGY" OR "OPHTHALMOLOGY" OR "ORTHOPEDICS" OR "OTORHINOLARYNGOLOGY" OR "PALEONTOLOGY" OR "PATHOLOGY" OR "PEDIATRICS" OR "PHARMACOLOGY PHARMACY" OR "PHYSIOLOGY" OR "PLANT SCIENCES" OR "PSYCHIATRY" OR "PUBLIC ENVIRONMENTAL OCCUPATIONAL HEALTH" OR "RADIOLOGY NUCLEAR MEDICINE MEDICAL IMAGING" OR "REHABILITATION" OR "REPRODUCTIVE BIOLOGY" OR "RESEARCH EXPERIMENTAL MEDICINE" OR "RESPIRATORY SYSTEM" OR "RHEUMATOLOGY" OR "SPORT SCIENCES" OR "SUBSTANCE ABUSE" OR "SURGERY" OR "TOXICOLOGY" OR "TRANSPLANTATION" OR "TROPICAL MEDICINE" OR "UROLOGY NEPHROLOGY" OR "VETERINARY SCIENCES" OR "VIROLOGY" OR "ZOOLOGY") OR TASCA==("ALLERGY" OR "ANESTHESIOLOGY" OR "AUDIOLOGY SPEECH LANGUAGE PATHOLOGY" OR "BEHAVIORAL SCIENCES" OR "CARDIAC CARDIOVASCULAR SYSTEMS" OR "CHEMISTRY MEDICINAL" OR "CRITICAL CARE MEDICINE" OR "DENTISTRY ORAL SURGERY MEDICINE" OR "DERMATOLOGY" OR "EMERGENCY MEDICINE" OR "ENDOCRINOLOGY METABOLISM" OR "ERGONOMICS" OR "ETHICS" OR "EVOLUTIONARY BIOLOGY" OR "FOOD SCIENCE TECHNOLOGY" OR "GASTROENTEROLOGY HEPATOLOGY" OR "GENETICS HEREDITY" OR "GERIATRICS GERONTOLOGY" OR "GERONTOLOGY" OR "HEALTH CARE SCIENCES SERVICES" OR "HEALTH POLICY SERVICES" OR "HEMATOLOGY" OR "IMMUNOLOGY" OR "INFECTIOUS DISEASES" OR "INTEGRATIVE COMPLEMENTARY MEDICINE" OR "MEDICAL ETHICS" OR "MEDICAL INFORMATICS" OR "MEDICINE GENERAL INTERNAL" OR "MEDICINE LEGAL" OR "MEDICINE RESEARCH EXPERIMENTAL" OR "NEUROSCIENCES" OR "NURSING" OR "NUTRITION DIETETICS" OR "OBSTETRICS GYNECOLOGY" OR "ONCOLOGY" OR "OPHTHALMOLOGY" OR "ORTHOPEDICS" OR "OTORHINOLARYNGOLOGY" OR "PALEONTOLOGY" OR "PARASITOLOGY" OR "PATHOLOGY" OR "PEDIATRICS" OR "PERIPHERAL VASCULAR DISEASE" OR "PHARMACOLOGY PHARMACY" OR "PRIMARY HEALTH CARE" OR "PSYCHIATRY" OR "PSYCHOLOGY" OR "PSYCHOLOGY APPLIED" OR "PSYCHOLOGY CLINICAL" OR "PSYCHOLOGY DEVELOPMENTAL" OR "PSYCHOLOGY EXPERIMENTAL" OR "PSYCHOLOGY MULTIDISCIPLINARY" OR "PSYCHOLOGY SOCIAL" OR "PUBLIC ENVIRONMENTAL OCCUPATIONAL HEALTH" OR "RADIOLOGY NUCLEAR MEDICINE MEDICAL IMAGING" OR "REHABILITATION" OR "REPRODUCTIVE BIOLOGY" OR "RESPIRATORY SYSTEM" OR "RHEUMATOLOGY" OR "SOCIAL SCIENCES BIOMEDICAL" OR "SUBSTANCE ABUSE" OR "SURGERY" OR "TOXICOLOGY" OR "TRANSPLANTATION" OR "TROPICAL MEDICINE" OR "UROLOGY NEPHROLOGY" OR "VIROLOGY")))

AND PY==("2016" OR "2017" OR "2018")

# Supplement 2 – Detailed inclusion and exclusion criteria

eTable 2. Further information on inclusion and exclusion of studies

| Include | Exclude |
| --- | --- |
| - Tool-development studies which focus primary on (specific) health outcomes - General Health studies (i.e. gender/sexuality-Studies, nutrition studies, psychological studies, pregnancy/parenthood)   With explicit focus on health outcomes or interventions.   - Rcts with embedded qualitative methods - Studies without clear focus on health outcomes but i.e. on family structure, caregivers, … in clinical settings and in health care - Studies describing an aspect of healthcare (connected to the above) - Educational Studies in clinical/health-service settings with i.e. improvement of training in health-professions. - Ecological studies with explicit (public)health impact (i.e. HIV prevention, mosquito-nets, …) - Public Health studies including qualitative research | - Validation studies - Tool-development studies which focus on tool-development (methodical) only - Educational studies investigating teaching methods - General studies without focus or mentioning on health outcomes or health interventions - Gender/sexuality-Studies without focus or mentioning on health outcomes - Research studies which focus on research/methodical aspects only - Studies that aimed at developing core outcome sets (only) - Studies in populations with a health condition or their relatives or care-givers with a focus on investigating an aspect of everyday-life without specific attention to the interaction with the condition or related interventions - Sociological studies i.e. on bullying without focus or mentioning on health outcomes or health interventions - Theoretical papers discussing aspects in health or research etc. |

# Supplement 3 – Further details for study and author characteristics

eTable 3. Definitions or examples of ambiguous characteristics of authors and studies that were used in the analyses

| Author and study characteristics | Categories | Definitions or Examples |
| --- | --- | --- |
| Gender of 1st author | Female, Male or Unclear | If the gender of the author was not immediately recognizable from the name, the individuals’ pictures were searched via google and institutional websites to enable a clear coding of information. If any uncertainties persisted, the assignment was categorized as 'unclear'. |
| Presentation format | Poster | There was explicit information in the database record or abstract that the abstract was presented as poster (print or digital) |
|  | Presentation | There was explicit information in the database record or abstract that the abstract was presented an oral presentation |
|  | Unclear | No information could be found |
| Affiliation category | Public | e.g. university, university hospital, public or non-profit institution |
|  | Private | e.g. private hospital, industry, private or for-profit institution |
| Level of healthcare addressed by the study | Tertiary care | e.g. hospital, ICU |
|  | Community care | e.g. home care |
|  | Secondary care | e.g. disease-specific medical care as rheumatoid arthritis or diabetic control |
|  | Primary care | e.g. general practitioner |
|  | Mixed | Study/Intervention focus/implementation was on different levels of care (e.g. hospital and home care) |
| Funding type | Public / non-profit | Funded by governmental or non-profit organizations |
|  | Private / for-profit | Funded by e.g. private, industrial, pharmaceutical companies or non-profit organizations |
|  | No funding | It was explicitly stated that the study/ research was not funded |
|  | Not reported | No information could be retrieved |
| Number of population groups included | Consecutive whole numbers | Population groups refer to groups in focus of each study such as professionals, nurses, patients, relatives or other. |

# Supplement 4 – Cox Regression: Characteristics associated with time-to-publication

eTable 4. Conference abstract characteristics and their association with time to publication

| factor | HR (95% CI) |
| --- | --- |
| Author characteristics |  |
| Gender of 1th author (female vs. male) | 0.92 (0.78-1.07) |
| Affiliation location |  |
| North America (reference category) | Not applicable |
| Europe | 1.15 (0.98-1.34) |
| Africa | 0.83 (0.44-1.56) |
| South America | 0.82 (0.40-1.65) |
| Asia | 2.07 (1.64-2.62)* |
| Australia | 1.91 (1.25-2.90)* |
| Affiliation category (private vs. public) | 0.69 (0.42-1.14) |
| Study characteristics |  |
| Presentation format (poster vs. presentation) | 0.60 (0.43-0.84)* |
| Number of population groups included | |
| 1 (reference category) | Not applicable |
| 2 | 1.02 (0.84-1.24) |
| >2 | 1.01 (0.75-1.38) |
| Number of qualitative data collection methods used | |
| 1 (reference category) |  |
| 2 | 1.05 (0.86-1.38) |
| >2 | 1.09 (0.70-1.71) |
| Level of healthcare addressed |  |
| Tertiary care (reference category) | Not applicable |
| Secondary care | 0.93 (0.73-1.17) |
| Primary care | 1.13 (0.86-1.48) |
| Community care | 1.35 (1.09-1.66)* |
| Mixed | 1.04 (0.67-1.62) |
| Funding reported (yes vs. no) | 1.21 (1.04-1.41)* |
| Funding type (private / for profit vs. public / non profit) | 0.79 (0.58-1.07) |
| Study aim reported (no vs. yes) | 1.38 (1.05-1.83)* |
| Study conclusions reported (no vs. yes) | 1.38 (1.05-1.83)* |
| Study type (cross-sectional vs. longitudinal) | 1.1 (0.85-1.43) |

Missing data and unclear information were excluded for the analysis of associations.

Abbreviation: HR, Hazard-Ratio; CI, confidence interval.

* statistically significant: p < 0.05

# Cox-Regression plots

eFigure 1. Plot of time to event analysis with gender of 1th author

eFigure 2. Plot of time to event analysis with affiliation location

eFigure 3. Plot of time to event analysis with affiliation category

eFigure 4. Plot of time to event analysis with presentation format

eFigure 5. Plot of time to event analysis with number of population groups included

eFigure 6. Plot of time to event analysis with qualitative data collection methods used

eFigure 7. Plot of time to event analysis with level of healthcare adressed

eFigure 8. Plot of time to event analysis with funding reported

eFigure 9. Plot of time to event analysis with funding type

eFigure 10. Plot of time to event analysis with study aim reported

eFigure 11. Plot of time to event analysis with study conclusions reported

eFigure 12. Plot of time to event analysis with study type
